# Walking autonomy in chronic dialysis patients: insights from a nationwide registry and focus group analysis

**DOI:** 10.1093/ckj/sfaf213

**Published:** 2025-08-07

**Authors:** Marine Naudin, Cécile Couchoud, Mathilde Lassalle, Nicolas Goin, Maud François, Marina Serru, Roxana Virdol, Alexandre Ganea, Delphine Dedenis, Ana Ferreira, Marion Delaporte, Matthias Buchler, Jean-Michel Halimi, Bénédicte Sautenet

**Affiliations:** Department of Nephrology, CHRU de Tours, Service de Néphrologie-Hypertension Artérielle, Dialyses et Transplantation Rénale, Tours, France; REIN Registry, Agence de La Biomedicine, Paris, France; Université de Tours, Université de Nantes, INSERM, methodS in Patient-centered outcomes and HEalth ResEarch (SPHERE), Tours, France; F-CRIN INI-CRCT (Cardiovascular and Renal Clinical Trialists), Nancy, France; REIN Registry, Agence de La Biomedicine, Paris, France; REIN Registry, Agence de La Biomedicine, Paris, France; Department of Nephrology, CHRU de Tours, Service de Néphrologie-Hypertension Artérielle, Dialyses et Transplantation Rénale, Tours, France; Department of Nephrology, CHRU de Tours, Service de Néphrologie-Hypertension Artérielle, Dialyses et Transplantation Rénale, Tours, France; Departement of dialysis, A.T.I.R.R.O. Orléans Association pour le Traitement des Insuffisants Rénaux de la Région Orléanaise; Departement of dialysis, A.T.I.R.R.O. Orléans Association pour le Traitement des Insuffisants Rénaux de la Région Orléanaise; Departement of dialysis, A.T.I.R.R.O. Orléans Association pour le Traitement des Insuffisants Rénaux de la Région Orléanaise; Departement of dialysis, A.I.R.B.P. Association des Insuffisants Rénaux Beauce Perche; Departement of dialysis, A.I.R.B.P. Association des Insuffisants Rénaux Beauce Perche; Departement of dialysis, A.I.R.B.P. Association des Insuffisants Rénaux Beauce Perche; Department of Nephrology, CHRU de Tours, Service de Néphrologie-Hypertension Artérielle, Dialyses et Transplantation Rénale, Tours, France; Department of Nephrology, CHRU de Tours, Service de Néphrologie-Hypertension Artérielle, Dialyses et Transplantation Rénale, Tours, France; F-CRIN INI-CRCT (Cardiovascular and Renal Clinical Trialists), Nancy, France; Department of Nephrology, CHRU de Tours, Service de Néphrologie-Hypertension Artérielle, Dialyses et Transplantation Rénale, Tours, France; REIN Registry, Agence de La Biomedicine, Paris, France; Université de Tours, Université de Nantes, INSERM, methodS in Patient-centered outcomes and HEalth ResEarch (SPHERE), Tours, France; F-CRIN INI-CRCT (Cardiovascular and Renal Clinical Trialists), Nancy, France

**Keywords:** chronic kidney disease, focus group, kidney replacement therapy, national registry, walking disability

## Abstract

**Background:**

Patients with chronic kidney disease (CKD) requiring kidney replacement therapy experience a loss in walking autonomy. This study used data from the French Renal Epidemiology and Information Network in Nephrology (REIN) registry and those from the National Health Data System [Système national des Données de Santé (SNDS)] to assess associations between walking inabilities and patient characteristics.

**Methods:**

We extracted data on all patients receiving kidney replacement therapy in France as of 31 December 2020. We used logistic regression to evaluate data associated with walking autonomy. We also created four focus groups to explore the perspectives of dialysis patients with respect to walking autonomy, and conducted a thematic analysis of qualitative data.

**Results:**

Data were available for 50 629 adults undergoing dialysis. Among the remaining 48 243 patients without missing data, 6834 (14%) were identified as having a walking disability, defined as walking with assistance or being totally unable to walk. Walking disabilities were associated with numerous comorbidities after adjustment for age. Despite their limitations, 2117 (46%) patients requiring assistance and 730 (43%) with total inability to walk had not been reimbursed for a mobility aid through the National Health Data System (SNDS). Thematic analysis identified four major themes from the focus groups: difficulties carrying out daily tasks, impact on social life, impact on connection to the body and psychological impact.

**Conclusion:**

Walking disability is strongly associated with patient comorbidities. It impairs quality of life, particularly through psychological and social consequences. Dedicated interventions and improved access to assistive devices are needed to help patients with walking disabilities.

KEY LEARNING POINTS **What was known:**

- A loss of autonomy in walking is observed in patients with chronic kidney disease.
- A correlation between loss of autonomy in walking and patient survival has been demonstrated.

**This study adds:**

- This study highlighted the challenges involved in caring for dialysis patients with walking difficulties and the psychological burden associated with impaired mobility.
- Our findings underscore the need to implement interventions to support patients with walking difficulties and to reduce the social and emotional consequences of impaired mobility.

**Potential impact:**

- In clinical practice, this study may improve our understanding of the impact of walking difficulties for dialysis patients, better inform dialysis patients regarding available assistive devices and support services, and better target patients material and psychological needs.

## INTRODUCTION

Patients with chronic kidney disease (CKD) are often physically inactive [1] and mobility tends to decline as the disease progresses [2]. Impaired mobility is closely linked to CKD, which is associated with physical inactivity, sedentary lifestyle and poor physical function [1, 3]. These factors contribute to adverse clinical outcomes, including an increased risk of mortality [4]. Besides, the higher the CKD stage, the more physical function declines [2]. The initiation of dialysis is associated with a substantial decline in functional status, especially among older adults [5]. A longitudinal study showed that overall gait speed is low in dialysis patients, and 54% continued to lose gait speed over 2 years [6]. Age, increased Charlson Comorbidity Index, C-reactive protein level and emergency dialysis start were found to be significant predictors of decreased walking independence in dialysis patients [7].

Furthermore, loss of autonomy among patients requiring kidney replacement therapy (RRT) has been associated with reduced survival. Low muscle strength has been associated with increased mortality [8], and declines in physical function have similarly been associated with higher mortality rates both in patients with CKD and in those requiring RRT. In contrast, greater muscle strength and higher levels of physical activity were associated with reduced mortality risk [9]. In one study, walking autonomy had the most weight in estimating the 6-month mortality prognosis for patients on chronic dialysis [10]. The walking autonomy item was proposed in two scores developed in France: to estimate the decision score for evaluating kidney transplantation [11] and the decision algorithm for dialysis treatment [12].

The objective of Phase 1 (quantitative) of the study was to analyse data from the French Renal Epidemiology and Information Network in Nephrology (REIN) registry to identify associations between walking inabilities and patient characteristics among dialysis patients and to correlate these data with those

from the National Health Data System [Systeme national des Données de Santé (SNDS)] on reimbursements for walking aids. The objective of Phase 2 (qualitative) of the study was to conduct focus groups with patients and caregivers to better understand difficulties related to walking problems and identify areas related to walking that are important to them.

## MATERIALS AND METHODS

This project has obtained the favourable opinion of ‘Ethics committee in human research’ (Reference number: 2024 78).

### Phase 1: Quantitative study

This was an observational, cross-sectional study based on two national databases.

#### Data sources

The REIN registry is a national database whose institutional support is the French Biomedicine Agency. Its objective is to ensure the epidemiological monitoring of all patients with end-stage kidney disease treated by RRT in France [13].

The SNDS contains detailed information on medical care consumption for almost the entire population of France [14].

Data from the REIN registry were linked to those from the SNDS via deterministic indirect matching. This method consists in identifying patients who are present in both databases by comparing shared information available in the two databases [15].

#### Population

The present study included all patients on chronic dialysis, regardless of type of dialysis (both haemodialysis and peritoneal dialysis), as of 31 December 2020, extracted from the French national REIN registry. Analysed data were collected during 2020 annual follow-up. We also extracted from the REIN registry data on walking status, dialysis treatment modalities, mode of transportation, age and 17 comorbidities (Table 1). We extracted all comorbidities available in the REIN database. In the REIN registry, the ‘walking’ item is completed by medical and paramedical (a professional, an allied health professional or a nurse) teams who are asked to indicate whether the patient is able to walk independently, unable to walk or able to walk with assistance.

**Table 1:** Patient characteristics by walking ability.

	*N* total = 50 628				
	Missing data = 2385				
	Independent walking	Walking with assistance		Total inability to walk	
	*N* = 41 409 (86%)	*N* = 5012 (10%)		*N* = 1822 (4%)	
Variables			OR^a^ (95% CI)		OR^a^ (95% CI)
Age group, years, mean (SD)	41 409 (86)	5012 (10)		1822 (4)	
18–44, *n* (%)	3328 (9)	84 (2)	0.2 (0.1; 0.3)	67 (4)	0.4 (0.3; 0.5)
45–64, *n* (%)	10 879 (27)	564 (11)	0.4 (0.4; 0.5)	350 (19)	07 (0.6; 0.8)
65–74, *n* (%)	11 443 (27)	1306 (26)		536 (30)	
>75, *n* (%)	15 559 (37)	3058 (61)	1.7 (1.6; 1.8)	869 (47)	1.1 (1; 1.3)
Body mass index, kg/m^2^, mean (SD)	26.5 (6.4)	27 (6.6)	1 (1; 1)	27 (7)	1 (1; 1)
Sex, female, *n* (%)	15 316 (37)	2500 (50)	1.7 (1.7; 1.7)	823 (45)	1.4 (1.4; 1.4)
Comorbidity, *n* (%)					
Diabetes	11 613 (28)	1956 (39)	1.8 (1.8; 1.8)	703 (38)	2.1 (2.1; 2.2)
Heart failure	5780 (14)	1118 (22)	1.9 (1.8; 1.9)	393 (21)	1.9 (1.9; 2.0)
Coronary insufficiency	7110 (17)	1176 (23)	1.4 (1.4; 1.4)	401 (22)	1.5 (1.5; 1.5)
Myocardial infarction	2837 (7)	458 (9)	1.3 (1.3; 1.4)	187 (10)	1.5 (1.5; 1.6)
Heart rhythm disturbances	6134 (15)	1108 (22)	1.7 (1.7; 1.7)	393 (21)	1.9 (1.9; 1.9)
Peripheral vascular disease	6107 (15)	1278 (25)	1.9 (1.9; 1.9)	538 (29)	3 (2.9; 3)
Amputation	316 (1)	249 (5)	6.5 (6.4; 6.6)	318 (18)	33.3 (32.7; 33.8)
Aneurysm	1046 (2)	136 (3)	1.1 (1.1; 1.1)	45 (2)	0.9 (0.8; 0.9)
Stroke, transient ischemic attack	3028 (7)	710 (14)	2.2 (2.2; 2.3)	304 (17)	2.7 (2.7; 2.7)
Hemiplegia	138 (0.3)	144 (3)	10.8 (10.6; 11.1)	247 (13)	48.3 (47.2; 49.4)
Respiratory failure	4455 (11)	704 (14)	1.4 (1.4; 1.4)	284 (16)	1.6 (1.6; 1.7)
Oxygen therapy	1780 (4)	325 (6)	1.4 (1.4; 1.5)	146 (8)	1.9 (1.9; 2.0)
Tobacco use	8334 (20)	930 (18)	0.5 (0.5; 0.5)	328 (18)	0.8 (0.8; 0.8)
Cancer	3088 (7)	416 (8)	1.1 (1.1; 1.1)	125 (7)	0.9 (0.9; 0.9)
Cirrhosis	627 (1)	89 (2)	1.1 (1.1; 1.2)	39 (2)	1.3 (1.3; 1.4)
Blindness	017 (2)	432 (9)	3.8 (3.7; 3.8)	139 (8)	3.9 (3.8; 4.0)
Behavioural disorder	812 (2)	276 (5)	3.2 (3.2; 3.3)	104 (6)	3.9 (3.9; 4.0)

Data are *n* (%) unless otherwise indicated.

^a^Compared with independent walking, adjusted on age and sex.

OR, odds ratio; 95% CI, 95% confidence interval.

The data retrieved from the SNDS were all reimbursements for mobility aids such as crutches and wheelchairs.

We explored the association between patient characteristics and walking status in three classes (independent walking, requiring another person or total inability to walk) with a multinomial logistic regression model adjusted for age and sex. Data were analysed using xl stat software 2016.3.

### Phase 2: Qualitative study

We selected the focus group method because it facilitates the exploration of a wide range of perspectives through interactive discussion. Group dynamics promote the emergence of individual experiences like a chain reaction thanks to the gathering of diverse personalities, which in turn encourages the expression and discussion of controversial opinions. Compared with individual interviews, focus groups may give greater prominence to critical or dissenting opinions, providing richer insight into shared concerns and tensions within the group context [16].

We conducted four focus groups with patients and caregivers. Data were collected from September 2022 to June 2023. The focus groups were led by a moderator. They took place in a quiet private room close to the dialysis units. We piloted a standardized question guide. Using open, circular and clarifying questions, participants were encouraged to reflect on and discuss their perspectives of loss of walking autonomy. All verbatim recordings were transcribed.

The inclusion criteria for participation in the focus group were: a current or past history of walking difficulties, regardless of severity; age ≥18 years undergoing chronic haemodialysis or peritoneal dialysis; and the ability to speak and understand French. For the first two focus groups, patients were recruited from hospital-based dialysis units and for the other two focus groups, patients were recruited from community-based dialysis units. Each participant received a non-opposition consent form regarding the use of their data. All patients agreed to the use of their data. The number of patients to be included in the focus groups was not determined in advance. We stopped at data saturation, which is the point at which collecting new data stops generating new ideas or themes. The focus groups were facilitated by a doctor of biology and research engineer from the nephrology department, in collaboration with a university professor and practicing nephrologist. Both were trained in focus groups and familiar with this patient population. The question guide was used as an aid. Additional questions might be used to adapt the discussion. We used a qualitative content analysis with an inductive approach to coding and subsequent theory development [17]. The data were processed according to the following steps, based on the method described in the reference article by Braun and Clarke (2006) [18]:

(i) Familiarization with our data: listening to the recordings to fully understand the content of the discussions, consideration of the initial data and transcription of the data by an external professional
(ii) Generation of initial codes: systematic coding of interesting features; each part of the transcript was categorized according to the idea it conveyed
(iii) Search for themes: grouping the codes into potential themes
(iv) Review of themes: verification of compatibility of themes with coded extracts
(v) Definition and naming of themes
(vi) Final analysis

#### Statistical analysis

Descriptive statistics were used for all variables of interest. Quantitative variables were presented as means with standard deviation (SD). Qualitative variables were expressed as numbers (percentages). The data distribution was assessed for the descriptive statistics. We used XL Stat software version 2016.3 for quantitative analyses. No software was used for the analysis of qualitative data.

## RESULTS

### Phase 1: Quantitative study and factors associated with walking difficulty

Among the 50 629 adults requiring dialysis patients in France as of 31 December 2020 registered in the REIN registry, 6834 (14%) walking with assistance (*n* = 5012) or were totally unable to walk (*n* = 1822) (Table 1). There were 37% women in the ‘independent walking’ group, 50% women in the ‘walking with assistance’ group and 45% women in the ‘total inability to walk’ group. Patients were older in the ‘walking with assistance’ group than in the independent walking and total inability to walk groups [mean (SD) age 76 (11.5), 68 (14.8) and 72 (13.2) years, respectively]. Body mass index was comparable among the groups.

The probability of walking with assistance was associated with age >75 years, increased body mass index and 16 of the 17 comorbidities (Table 1). Comorbidities associated with walking disability are peripherical vascular disease, having an amputation, a history of stroke, diabetes and heart failure. The probability of total inability to walk was associated with age >75 years, increased body mass index and 14 of the 17 comorbidities. The probability of walking with assistance was inversely associated with being a former or current smoker, and the probability of total walking disability was inversely associated with having an aneurysm, cancer or being a former or current smoker.

#### Walking aid prescription data (SNDS)

In total, 2117 (46%) of patients classified as ‘walking with assistance’ and 730 (43%) with ‘total inability to walk’ in the REIN register did not benefit from any SNDS device reimbursement (Table 2). Conversely, 3941 (10%) patients classified as ‘independent walking’ in the REIN register were reimbursed for a heavy device (i.e. walkers or vehicles for the physically disabled) from the SNDS.

**Table 2:** Pairing of the REIN registry and SNDS data by walking ability.

		REIN group, *n* (%)		
		Independent walking	Walking with assistance	Total inability to walk
SNDS ranking	Light device (crutches/canes)	6435 (17)	1156 (25)	394 (24)
	Heavy device (walker/vehicle for physically disabled patients)	3941 (10)	1334 (29)	566 (33)
	No reimbursement	27 221 (73)	2117 (46)	730 (43)

### Phase 2: Qualitative study

We created four focus groups: two in a hospital-based facility and two in an out-centre facility. We included a total of 15 patients (8 women; mean age 70 years) and 4 caregivers. The first focus group consisted of three patients. The second focus group consisted of four patients. The third focus group consisted of five patients and one caregiver. The fourth focus group consisted of three patients and three caregivers. There were few caregivers, and the analysis of their results was not possible because data saturation was not reached.

**Figure 1:**
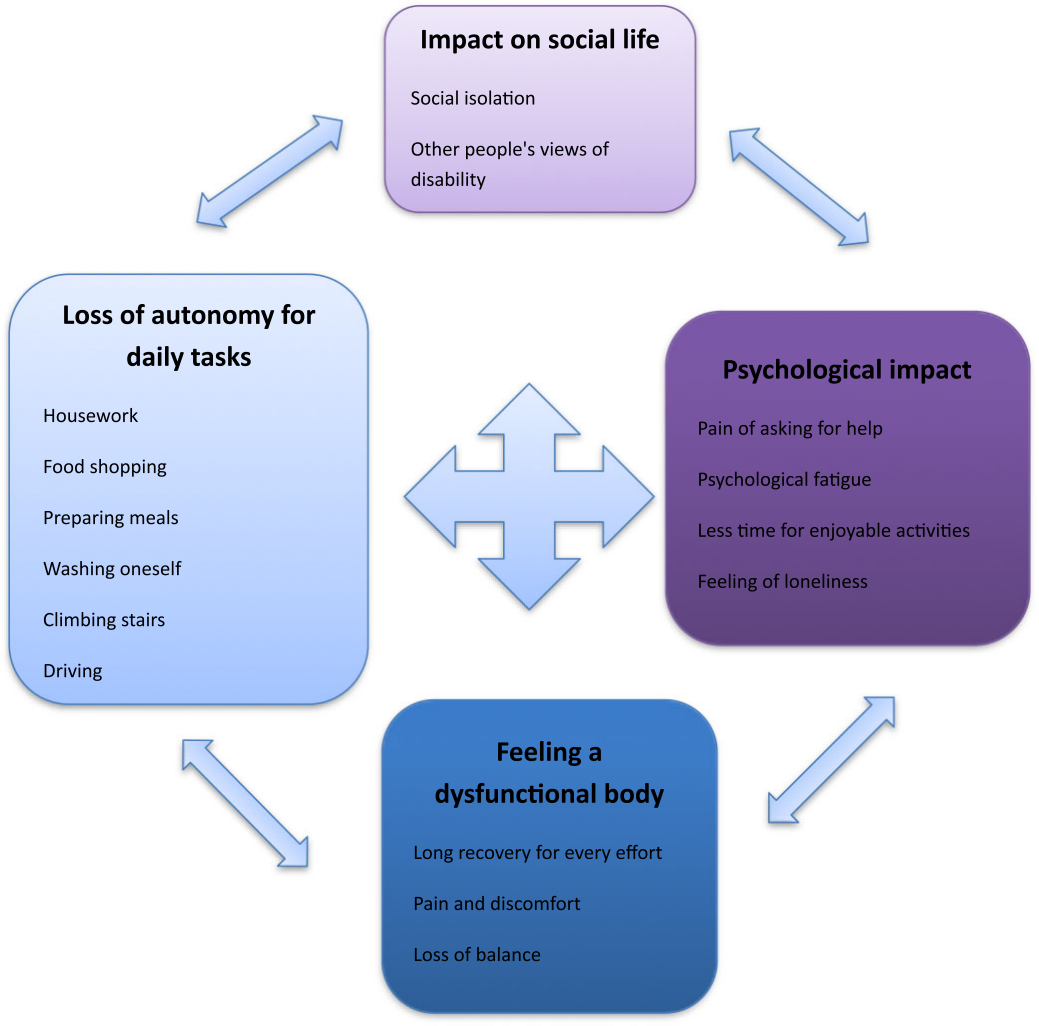
Categories and subcategories of themes of difficulties related to walking for patients on chronic dialysis.

The participants described all the problems they encountered related to walking, ranging from practical difficulties in everyday life to social and psychological concerns. Patients highlighted four themes: loss of autonomy in daily tasks, feeling a dysfunctional body, impact on social life and psychological impact. Each theme contained subcategories described in Fig. 1.

#### Loss of autonomy in daily tasks (Table 3)

The first theme participants mentioned concerned difficulties in daily tasks due to loss of autonomy in walking. This resulted in difficulties in six areas. First, patients mentioned difficulties with household chores. Patients explained this difficulty by increased slowness: ‘*The simple act of vacuuming took me 2 hours*.’ Patient 6, female, focus group 2, 50 years old.

Patients also had difficulty shopping, a task they considered one of the most important daily tasks because it allows them to eat. The patients expressed more difficulties on dialysis days for their daily activities with increased fatigue. The patients also spoke of difficulties in preparing meals and washing themselves or even climbing the stairs. Finally, patients spoke of the impact of the walking difficulty on driving.

One patient spoke about the link between no longer being able to drive and the social isolation he felt. He lives in the countryside and cannot see his children and grandchildren. Patients also spoke of the efforts required to overcome these difficulties and the need for great adaptation skills: ‘*I made a lot of efforts to return to an almost normal life, I made a lot of efforts*’. Patient 2, female, focus group 1, 76 years old.

#### Feeling of having a dysfunctional body (Table 3)

The second theme mentioned concerned the connection to the body. This was reflected in three areas. Several participants cited walking fatigue linked to difficulty walking long distances.

‘*For long distance travel, recovery is… much longer too*.’ Patient 5, male, focus group 2, 49 years old.

The second area concerned pain and discomfort.

The third area concerned loss of balance and fear of falling.

#### Impact on social life (Table 3)

The third theme identified was the impact of loss of autonomy on social life. Patients spoke of their social isolation.

The difficulty of how others view disability was also mentioned (Table 3).

**Table 3:** Patient quotations during focus groups.

Loss of autonomy in daily tasks
• ‘The simple act of vacuuming took me 2 hours.’ (Focus group 2, patient 6, female, 50 years)
• ‘And then, what we did in an hour, it takes 3 hours to do.’ (Focus group 3, patient 12, female, 77 years)
• ‘The fact of not having autonomy, of no longer driving, I suffer a lot from that.’ (Focus group 2, patient 5, female, 49 years)
• ‘I made a lot of effort to return to an almost normal life, I made a lot of effort.’ (Focus group 1, patient 2, female, 76 years)
• ‘but I see that sometimes, when I get to the checkout, uh... I... pfff... I can't take it anymore.’ (Focus group 2, patient 6, female, 50 years)
Feeling of having a dysfunctional body
• ‘For long distance travel, recovery is… much longer too.’ (Focus group 2, patient 5, female, 49 years)
• ‘I have difficulty changing position, I have pain.’ (Focus group, patient 10, female, 56 years)
• ‘It's spinning, there's a lack of strength in the legs, all that, the cramps, that's unpleasant.’ (Focus group 2, patient 4, male, 71 years)
• ‘So, I had a long period where I was falling over in my kitchen, and at one point I said to myself ‘my God’ because I didn't have the strength to get up...’ (Focus group 3, patient 8, female, 85 years)
• ‘I go down the stairs one step at a time, like this. I don't go down them like a normal person anymore.’ (Focus group 4, patient 15, female, 66 years)
Impact on social life
• ‘I don't go out.’ (Focus group 1, patient 1, male, 69 years)
• ‘I have a sign in front of my house… a disabled sign, people don't respect it, they park in front, in my space, that's it.’ (Focus group 2, patient 7, female, 67 years)
• ‘A person who doesn't walk very well, he is confined a bit at home... he can't contact people, it's very restricted.’ (Focus group 3, patient 11, male, 77 years)
• ‘You are pigeonholed.’ (Focus group 2, patient 7, female, 67 years)
• ‘There is one thing though. Those who are in the city have a little more help, if you like. For means of transport, they can travel by bus, they can still get around. In the countryside, there is nothing left. It is zero.’ (Focus group 4, patient 14, male, 85 years)
Psychological impact
• ‘Last summer, there was washing in bed. As soon as I was able to... take responsibility for myself too, it was important.’ (Focus group 1, patient 2, female, 76 years)
• ‘I feel like I've been emptied from the inside.’ (Focus group 2, patient 5, female, 49 years)
• ‘It's a lot of work for the spouse.’ (Focus group 3, patient 12, female, 82 years)
• ‘It's certain that being alone for eight or ten years seems like a long time.’ (Focus group 4, patient 14, male, 85 years)

‘*I have a sign in front of my house… a disabled sign; people don't respect it, they park in front, in my space, that's it*.’ Patient 7, female, focus group 2, 67 years old.

#### Psychological impact (Table 3)

The fourth theme cited was the psychological impact of loss of walking autonomy. The difficulty in asking for help and the relief of regaining autonomy was mentioned.

Psychological fatigue, to the extent of psychological exhaustion, was also mentioned: ‘*I feel like I've been emptied from the inside*.’ Patient 5, male, focus group 2, 49 years old.

The hedonic dimension was also cited, referring to reduced time available for pleasant activities, with increased burden on the caregiver.

Finally, participants expressed feelings of loneliness.

## DISCUSSION

Our study confirms the association between the inability to walk and the majority of comorbid conditions in patients receiving dialysis, which supports the importance of walking autonomy as a marker of disease severity in this population. In addition, few patients used walking devices. Walking impairment was linked to the perception of a reduced quality of life for dialysis patients.

The link between walking disorders and arteritis is not surprising. Symptoms of claudication have often been reported in patients with arteritis [19]. Said *et al*. described threatened gait safety in patients with stroke [20]. Patients with heart failure commonly report symptoms of reduced functional ability [21]. These results show the consistency of the data from the REIN registry.

The link between walking difficulties and sex cannot be explained by age. Indeed, the analysis was adjusted for age. However, for men and women at the same age, older women tend to fall more often than do men [22]. A study demonstrated that older women showed impairments in balance [23]. The authors explain this difference by biomechanical origins (dorsiflexion strength and range of motion). Furthermore, female sex is a risk factor for fear of falling, a predictor of falling [24]. This finding could explain why female sex is a risk factor for walking difficulty.

The walking behaviour disorders we observed in dialysis patients may be linked to the presence of a psychological disorder (depression, psychotic disorders) or dementia. In older people, several studies have suggested a link between walking disabilities and dementia. For example, strong associations have been found between slow walking pace and decline in executive functions [25]. Gait impairments and falls are more prevalent in people with dementia than in normal aging individuals and are related to severity of cognitive impairment [26, 27]. Impaired cognitive abilities can reduce attentional resource allocation, which can compromise postural and gait stability [28].

Inconsistent results concerning the fact that having cancer and aneurysm would seem to be protective against walking difficulties may be explained by the survival bias of the analysis of patients. Indeed, those who were in poor health had already died [29].

A total of 46% of patients classified as ‘walking with assistance’ and 43% of those with total inability to walk in the REIN registry did not benefit from any SNDS reimbursements for devices.

In the thematic analysis, we found difficulties in carrying out daily tasks such as cleaning one's home, shopping, cooking, cleaning oneself, walking long distances, climbing stairs and driving as major obstacles in the life of chronic dialysis patients. The focus groups allowed us to understand that these difficulties are linked to a great slowness, to which is added fatigue from dialysis days, along with difficult recovery from exercise. Frailty has been described in dialysis patients. This syndrome is characterized by decreased ability to resist health stressors and a vulnerability to adverse health consequences [30]. This syndrome could be explained by reduced strength and diminished physiological reserve. The frailty could affect difficulties in daily tasks. Added to this, patients described difficulty seeking help. They described the burden the frailty [31] placed on their caregivers. Also, patients with RRT must demonstrate great adaptation skills (adapting to doing things more slowly, sometimes changing accommodation or giving up certain activities that they found enjoyable). Patients often described the efforts required for this adaptation during interviews.

Patients’ connection to their body was altered. This results in a painful body, which recovers more slowly and in which patients have less confidence. A meta-analysis performed in 2020 revealed themes concerning the experience of pain in dialysis patients, including agony, suffering in silence, provoking fear of treatment and preventing life participation [32]. Patients also mentioned the fear of falling, defined as a lasting concern about falling that ultimately results in a restriction of daily life activities [33]. Fear of falling can constitute a major obstacle when we consider that the physical activity of patients is essential for their recovery and even directly linked to their survival [30, 31]. This fear of falling can cause patients to practice less physical activity, which in turn causes further deterioration.

These patients also experience social isolation, particularly when they live in the countryside and/or far from their loved ones. Because of the disability of these patients, combined with the fatigue of dialysis and the scrutiny of others, they are vulnerable to social isolation. This latter is also predictive of a greater risk of cognitive decline [28], cardiovascular disease [34], cerebrovascular disease [35] and mortality [36]. A study from China showed that social isolation is significantly associated with kidney function decline and the development of CKD [37]. The authors explained this correlation by individuals not socially isolated having better healthcare knowledge [38]. In addition, isolated people demonstrate poorer compliance with treatments [39]. From a physiological perspective, social isolation may worsen the pro-inflammatory state of CKD [40] and is linked to lower serum albumin levels [41]. Social isolation undermines patient mental health, particularly self-esteem and sense of belonging [42]. Preventing this social isolation, which can be mediated by a professional psychologist, is crucial for care teams.

These different areas are also self-sustaining because difficulties in daily activities affect social life and then the psychological sphere. The example of driving clearly illustrates this vicious circle. Indeed, no longer being able to drive can result in social isolation and can have a psychological impact (depression).

The strengths of our study are the use of linked national registries and the combination of quantitative descriptive and qualitative analyses. Our work has also some limitations. First, the people who participated in focus groups were probably the most motivated and the most available (not necessarily representative of all chronic dialysis patients with walking difficulties). Second, we had few caregivers in our focus groups, and their perception could have enhanced our results. Third, there are potential biases related to missing data.

Our results highlight the complex challenges involved in managing dialysis patients with walking difficulties, particularly the psychological burden associated with these limitations. Furthermore, they demonstrate the importance of implementing interventions to help patients with walking difficulties. This work presents several avenues for future research. First of all, it would be interesting to observe the link between walking behaviour disorders observed in dialysis patients and possible psychological disorders (depression, psychotic disorders). Moreover, our data suggest the importance of the medical team reminding dialysis patients of the available device aids covered by the SNDS and of scheduling an appointment with a social worker if necessary to improve patient information. Furthermore, the administrative slowness of obtaining a device aid should be explored to understand this problem. To support patients, medical and paramedical teams could develop interventions aimed at helping patients suffering from walking disorders. This could result in non-medical interventions such as yoga, sophrology or meditation. In addition, sport or physiotherapy during dialysis sessions could be offered to patients. Finally, it would be interesting to recall the material aids available (advice from social services) or even the help of a psychologist.

## Data Availability

Data available on request.
